# Femoral press-fit fixation in ACL reconstruction using bone-patellar tendon-bone autograft: results at 15 years follow-up

**DOI:** 10.1186/1471-2474-13-115

**Published:** 2012-06-27

**Authors:** Wojciech Widuchowski, Malgorzata Widuchowska, Bogdan Ko czy, Szymon Dragan, Andrzej Czamara, Wieslaw Tomaszewski, Jerzy Widuchowski

**Affiliations:** 1 District Hospital of Orthopedics and Trauma Surgery, Department of the Knee Surgery, Arthroscopy and Sports Traumatology, Piekary Slaskie, Poland; 2 Department of Internal Medicine and Rheumatology, Medical University of Silesia, Katowice, Poland; 3 Department and Clinic of Orthopaedic and Traumatologic Surgery, Faculty of Postgraduate Medical Training, Wrocław Medical University, Wrocław, Poland; 4 College of Physiotherapy in Wrocław, Wrocław, Poland

## Abstract

**Background:**

If anterior cruciate ligament (ACL) reconstruction is to be performed, decision regarding graft choice and its fixation remains one of the most controversial. Multiple techniques for ACL reconstruction are available. To avoid disadvantages related to fixation devices, a hardware-free, press-fit ACL reconstruction technique was developed.

The aim of this study was to evaluate clinical outcome and osteoarthritis progression in long term after ACL reconstruction with central third patellar-tendon autograft fixed to femur by press-fit technique.

**Methods:**

Fifty two patients met inclusion/excusion criteria for this study. The patients were assessed preoperatively and at 15 years after surgery with International Knee Documentation Committee Knee Ligament Evaluation Form, Lysholm knee score, Tegner activity scale and radiographs.

**Results:**

Good overall clinical outcomes and self-reported assessments were documented, and remained good at 15 years. The mean Lysholm and Tegner scores improved from 59.7 ± 18.5 and 4.2 ± 1.0 preoperatively to 86.4 ± 5.6 (*p* = 0.004) and 6.9 ± 1.4 (*p* = 0.005) respectively at follow-up. The IKDC subjective score improved from 60.1 ± 9.2 to 80.2 ± 8.1 (*p* = 0.003).

According to IKDC objective score, 75% of patients had normal or nearly normal knee joints at follow-up. Grade 0 or 1 results were seen in 85% of patients on laxity testing. Degenerative changes were found in 67% of patients. There was no correlation between arthritic changes and stability of knee and subjective evaluation (*p* > 0.05).

**Conclusions:**

ACL reconstruction with patellar tendon autograft fixed to femur with press-fit technique allows to achieve good self-reported assessments and clinical ligament evaluation up to 15 years. Advantages of the bone-patellar-tendon-bone (BPTB) press-fit fixation include unlimited bone-to-bone healing, cost effectiveness, avoidance of disadvantages associated with hardware, and ease for revision surgery. BPTB femoral press-fit fixation technique can be safely applied in clinical practice and enables patients to return to preinjury activities including high-risk sports.

## Background

The anterior cruciate ligament (ACL) is regarded as critical to the normal functioning of the knee, and it is one of the most frequently injured ligaments in the human body. Its rupture affects knee stability, which may cause giving way symptoms, increased risk of meniscal injuries, and early onset of joint degeneration [1-5].

When treating a torn ACL, many decisions must be made, especially if surgery is to be performed. The decision regarding graft choice and its fixation remains one of the most controversial. The graft could be autograft, allograft, or synthetic. These include patellar tendon, hamstring tendons, quadriceps tendon and others [4,6-11].

Central third bone–patellar tendon– bone autograft fixed with interference screws has long been the graft of choice (especially when dealing with athletes involved in contact sports) [12-14], despite certain number of various complications that have been reported [15-18].

To avoid disadvantages related to internal fixation devices, especially on femoral side, a hardware-free ACL reconstruction technique was developed. This technique uses the bone plugs on either end of the patellar tendon graft for press-fit fixation. The presented technique was originally developed in 1987 for femoral press-fit fixation and in 1989 for tibial press-fit fixation [19,20]. Afterwards it was used and popularized by other authors [21-24]. The press-fit fixation was reported to have a similar pull-out strength and stiffness when compared to hardware fixations [24-26] and accepted as an effective and cost reducing method for ACL reconstruction.

The aim of this retrospective study was to evaluate the clinical outcome and osteoarthritis progression in the long term after ACL reconstruction with a central third patellar-tendon autograft fixed to the femur by press-fit technique.

## Methods

### Inclusion criteria

The entry inclusion criterion for this study was isolated ACL insufficiency combined with subjective knee instability. Patients who had had a previous injury or surgery on either knee, patients with bilateral ACL insufficiency; a posterior cruciate ligament (PCL) insufficiency; an injury of postero-lateral corner (PLC), lateral collateral ligament (LCL), or medial collateral ligament (MCL) tear of grade III [27] were excluded. Patients with concurrent osteoarthritis, meniscal lesions, focal Outerbridge [28] grade III or IV cartilage lesions, were not included as well. We also excluded patients who had graft rupture, required revision or other surgery of the analysed knee or had ACL injury of the contralateral knee during follow-up period.

### Patients

Seventy one patients met the entry inclusion criteria. The analyzed group consisted of 41 men and 30 women. The average patients’ age was 28 (range: 16–43 years). The mean period between the initial injury and surgery was 3.2 months (range, 0.5 - 9.3 months). The BMI in the study group was 27.9 ± 3.8 kg/m^2^.

### Surgical technique

**Figure 1:**
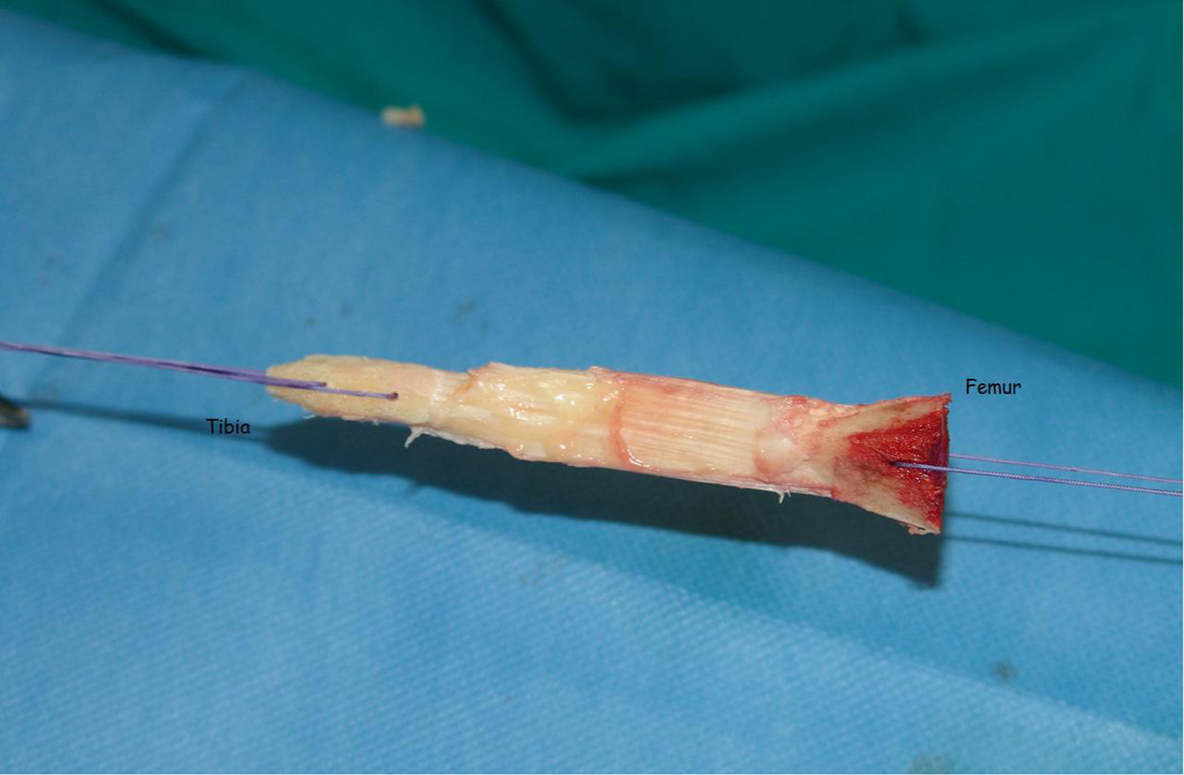
**The bone blocks aree formed to a trapezoid shape by using an oscillating saw.** The tibial bone block must be sized so that its basis can pass through tunnel of 10-11 mm diameter and rest of the bone block 9–10 mm diameter.

The miniarthrotomy transtibial technique using femoral press-fit fixation and tibial fixation with interference screw was performed. A diagnostic arthroscopy was carried out first. The middle third of the patella tendon (9–10 mm in diameter) was harvested with 25 mm to 30 mm of bone from the patella and tibial tubercle. The bone blocks were formed to a trapezoid shape by using an oscillating saw (Figure 1). The tibial bone block must be sized so that its basis can pass through a tunnel of 10-11 mm diameter and the rest of the bone block 9–10 mm diameter. Two 1.5-mm holes were drilled into each bone block. Through the donor site defect a mini-arthrotomy was then made.

**Figure 2:**
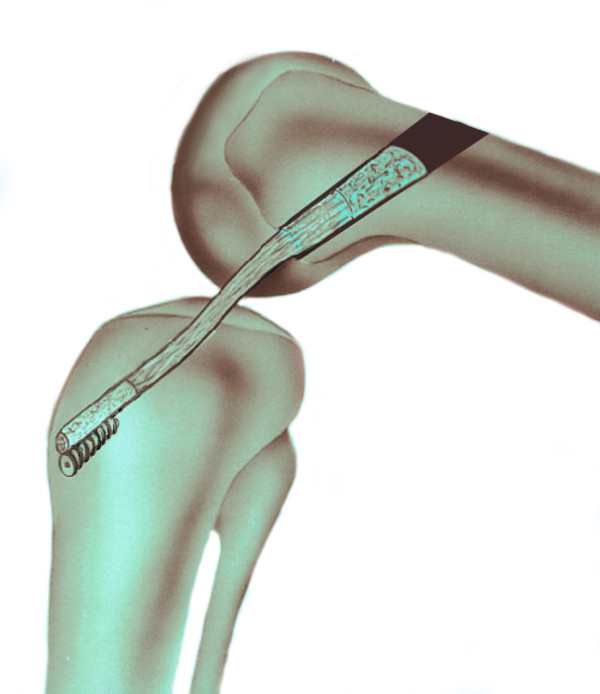
The tibial bone plug is tapped into the femoral tunnel and fixed with press-fit. The patellar bone plug is secured in the tibial tunnel with an interference screw.

The tibial tunnel was created using a drill guide inserted through previously made mini-arhtrotomy in a standard fashion into the posterior third of the native ACL insertion with the same hollow reamer. The length of the tibial tunnel was usually 45–50 mm. The femoral tunnel was drilled with an 9–10 mm hollow reamer from outside-in from the lateral aspect of the distal part of the femur (through a separate incision) to the ten-thirty position (for right knees) or the one-thirty position (for left knees) at the back of the intercondylar notch. The graft was then passed into the knee from outside-in using a pull-through suture and the bone blocks positioned in their tunnels by pulling and assisted with hammering using impactor inserted through a separate incision on thigh with the knee joint flexed up to 120°. The tibial bone plug was tapped into the femoral tunnel and fixed with press-fit. The patellar bone plug was secured in the tibial tunnel with an interference screw (Figure 2).

The insertion of the bone block was controlled by pulling the graft in the distal direction at the 10–20° position of the knee. All bones were harvested and filled to the patella and the tibial harvesting defect. After manual laxity was evaluated, the patellar tendon, paratenon, subcutaneous tissue, and skin were closed.

### Rehabilitation

All patients underwent the same rehabilitation program after the surgery.

#### 1–3 week

A fixed splint in full extension was worn in the first week. The patient walked with toe touch weight-bearing using crutches. The immediate active quadriceps isometric exercises were started. On the tenth postoperative day, the brace was adjusted to allow motion between 0° and 60° of flexion. The patient continued walking with toe-touch weight-bearing using crutches.

#### 3–6 week

Three weeks after surgery, the brace was adjusted to allow between 0° and 90° of flexion and the patient was permitted to bear weight as tolerated without crutches while wearing the brace. At four weeks, use of the brace was discontinued and full weightbearing was allowed as tolerated. Full isotonic hamstring contraction, hip abductor-adductor exercises, and swimming were permitted. Six weeks after surgery, full flexion was allowed. The patient was allowed to ride a stationary bicycle without resistance.

#### 6–12 week

At eight weeks, patients were encouraged to achieve a full range of motion, to extend the knee against unlimited resistance and to ride a stationary bicycle with resistance. At twelve weeks, unrestricted isotonic quadriceps-strengthening was allowed between 0° and 90° of flexion. The patient was allowed to ride a bicycle outdoors and to jog at half speed.

#### 2–6 month

Two to 3 months after surgery, patients were allowed to ride a bicycle outdoors, to jog on solid ground, and to swim. At four months, running in a straight line was allowed and the first isokinetic strength-test was performed. Between six and eight months, the patient was allowed to return to sports if he had 90% isokinetic strength compared with that of the contralateral knee, no effusion, and a full range of motion. Return to pivoting and contact sports was allowed after 6 months if there were no effusion, full range of motion, and a muscle strength of 90% compared with the contralateral side.

### Follow-up evaluation

Patients were evaluated using the International Knee Documentation Committee (IKDC) score [29] (subjective and objective criteria), Lysholm score [30] and Tegner score [31]. Each patient was assessed preoperatively, and postoperatively at 15 years.

Laxity in both groups was tested with the Lachman [32] and pivot-shift [33] tests. Instrumented knee testing was performed using the Rolimeter (Aircast Europa, Neubeuern, Germany) at maximal manual force and a knee flexion angle of 20°, and compared with that of uninjured knee. The active and passive ranges of knee motion were measured.

Knee function was assessed with the one-legged-hop test (evaluated preoperatively and at 15 years), difficulty with performing the duckwalk, and difficulty with squatting. Stiffness of the knee, catching and locking of the knee, and anterior knee pain as well as extent of donor- site morbidity were also recorded.

Besides the parameters of the IKDC subjective score, we used other subjective outcome questions. “Would you have surgery again”, “Does your knee give way”

Radiographic evaluation was performed according to IKDC evaluation form and included a standing weight-bearing anteroposterior view (45° of knee flexion) [34] and lateral view.

The clinical evaluations were performed by a physician not involved in the primary ACL reconstruction.

### Statistics

The data was stored on a Microsoft Access database. Statistical analysis was performed using the Statistica 6 (StatSoft Inc) software program by medical statistician. Following tools were used: Pearson's chi-square test (*χ*2), the Mann–Whitney *U* test, Fisher’s exact test, the Pearson’s correlation coefficient and the Spearman's rank correlation coefficient. Significance was set at p < 0.05.

## Results

### Follow-up assessment

52 of initial 71 patients, according to inclusion/exclusion criteria, completed the 15-year follow-up (13.8.-16.2).

5 patients were excluded because of an ACL injury of the contralateral knee, 4 patients (5.6% of 71) had a graft rupture due to an adequate trauma during the follow-up period, 4 patients were lost to follow-up. In 8 patients, during follow-up period, further surgeries to address the secondary restraints were conducted (Table 1). Two patients were operated twice. In 3 cases more than one procedure was performed during the surgery.

**Table 1:** Complications and further surgeries in patients within both groups

**Surgery**	**N**	**%**
ACL graft rupture	4	6%
reACL reconstruction	3	4%
contralateral ACL rupture	5	7%
debridement	4	6%
loose body removal	3	4%
meniscectomy	5	7%
contralateral meniscectomy	3	4%
removal of screws	6	8%
stiffness requiring manipulation under anesthesia	2	3%
cartilage repair	3	4%

### Intraoperative findings

There were 24 patients with a grade I and 4 patients with a grade II instability of the MCL. 19 patients had damage to the tibial, femoral, and patellar articular cartilage of Outerbridge grade I or II.

### Clinical assessment

#### Range of movement

The median range of motion for the index limb was 0° of extension (−2° to 5°) to 131° of flexion (125 to 145). When compared with the contralateral limb, 17 of 52 (33%) patients had less than 5° of flexion loss, and 6 patients (11.5%) displayed between 5° and 15° of flexion loss. A flexion deficit of >15° was observed in 2 patients (3.8%). 7 patients (13%) had lost extension of 3°–5°, and in 3 patients (6%) an extension deficit of >5° was observed.

#### Ligament testing

##### Instrumented Testing

The mean anteroposterior translation measured with Rolimeter was 1.6 mm ± 1.3(a difference to the opposite knee). Instrumented test results were not associated with self-reported knee function (*p* = 0.07).

##### Pivot-Shift test, Lachman test

At 15 years, 81% and 83% of 52 patients had grade 0 and “+” Lachman and Pivot-shift test results, respectively (Table 2). There was no correlation between both Lachman and Pivot-shift tests and self-reported knee function (p = 0.08 and p = 0.1, respectively).

**Table 2:** The Lachman and Pivot-Shift tests at 15 years follow-up

**test/result**	**0**	**+**	**++**	**+++**
Pivot-Shift	7 (13%)	36 (70%)	8 (16%)	1 (2%)
Lachman	17 (33%)	29 (56%)	6 (11%)	0 (0%)

#### International knee documentation committee

Assessment using the overall IKDC objective score revealed that 75% of the patients had normal or nearly normal knee joints at follow-up (Table 3).

**Table 3:** IKDC objective score

	**A**	**B**	**C**	**D**
preoperatively	0 (0%)	3 (6%)	25 (48%)	24 (46%)
15-year FU	18 (35%)	22 (42%)	8 (15%)	4 (8%)

#### Activity level

Activity level was recorded with the use of the Lysholm score, Tegner score, and the subjective IKDC. The activity grade improved significantly over time (p = 0.01). Thirty nine of 52 patients (75%) returned to their previous sport at the preinjury level, including high-risk sports such as soccer or skiing (Table 4).

**Table 4:** Lysholm, Tegner and IKDC subjective scores at 15 follow-up

	**Lysholm**	**Tegner**	**IKDC subjective**
preoperatively	59.7 ±18.5	4.2 ± 1.0	60.1 ± 9.2
postoperatively	86.4 ± 5.6	6.9 ± 1.4	80.2 ± 8.1
*p* value	0.004	0.005	0.003

#### Functional assessment

The patients were asked to perform a single-legged hop for distance on the index and normal side. According to IKDC objective score a ratio of the index to normal knee was calculated: ≥90% (62% of cases), 89-76% (25% of cases), 75-50% (13% of cases). At the 15-year follow-up, 43 (83%) patients could squat normally, 7 (13%) others could do it with slight difficulty and 2 patients (4%) could squat with significant difficulty.

#### Patients satisfaction

When asked, 49 (95%) patients would have the surgery again. Subjectively, 2 patients that were seen for follow-up complained about instability. Anterior knee sensitivity (42% of patients reported the presence of kneeling pain) and donor site morbidity were the most often mentioned complaint. Numbness of the skin was reported by 37 (42%) patients.

### Radiographic evaluation

Radiographs were performed on 52 patients at 15 years. Degenerative changes were found in 37 (67%) patients. The results of the IKDC radiographic assessment are shown in Table 5. There was a significant increase of incidence and severity of osteoarthritis between preoperative assessment and 15 years (p = 0.001). In the medial tibiofemoral joint 38% of patients were graded as normal, 48% as nearly normal and 14% as abnormal. In the lateral tibiofemoral joint 59% of cases were graded as normal, 38% as nearly normal and 3% as abnormal. In the patellofemoral joint 65% of patients had no degenerative change (normal), 30% were graded as nearly normal and 5% as abnormal.

**Table 5:** IKDC Radiographic ratings at 15 years follow-up

	**normal**	**nearly normal**	**abnormal**	**severely abnormal**
preoperatively	32 (61.5%)	20 (38.5%)	0 (0%)	0 (0%)
15-year FU	17 (33%)	28 (54%)	7 (13%)	0 (0%)

There was no correlation between radiographic changes and postoperative results in both subjective and objective scores (Table 6). Ectopic bone formation was observed in 17 of the 52 patients (33%). It was located intraligamentous in the quadriceps tendon (7 cases), at the apex of the patella (6 cases), and at the proximal pole of the patella (4 cases).

**Table 6:** Correlation between radiographic changes and postoperative results

**test/ score**	**Lachman test**	**Pivo-shift test**	**IKDC subjective**	**Tegner score**	**Lysholm score**
*p* value	0.08	0.09	0.06	0.05	0.06

### Complications

Postoperative complications were observed in 5 patients. One patient developed superficial wound infection, which resolved on treatment with antibiotics. Arthrofibrosis occurred in 4 patients, requiring intensive treatment, in 2 cases arthroscopical arthrolysis. There were 3 late arthroscopies, for arthrolysis to allow full extension.

## Discussion

The success of the ACL reconstruction is influenced by different factors. One of them is the choice of the graft. It is clear that an ideal graft for ACL reconstruction does not exist. For many surgeons, bone–patellar tendon– bone autograft (BPTB) remains still the graft of choice. Although, the graft is criticized for resulting in significant harvest-site morbidity, over the years it has proved to be a stable graft that has longlasting biomechanical properties [3,14,15,35,36]

The primary stability of the graft is one of the most important issues in ACL reconstruction. It depends not only on the strength of the graft, but also on its fixation. Regarding BPTB, the graft can be fixed using different methods of fixation e.g.: interference screw, button, staple post and others. It can also be fixed with the use of press-fit technique without use of any hardware [4,20,22,25,37].

The femoral press-fit fixation in this study utilizes the native femoral ACL insertion as the point of fixation. The graft is protected additionally by the angulation of the bone block against the ligament graft thereby also minimizing the risk of tunnel misplacement or bone tunnel enlargement [20]. The most important advantage of press-fit technique is a stable bony fixation on the femoral site without interference screw. It enables direct bone to bone healing and reduces the number of disadvantages associated with hardware fixation, like the inadvertent graft advancement, graft laceration, bone resorption or chronic synovitis, allergic reactions [21,23,24]. Another important feature of this fixation technique is easier revision of ACL reconstruction [20,37]. The results of any kind of ACL reconstruction technique should also be evaluated from an economic point of view. As it was documented by Forssblad et al. [38], patellar tendon graft is superior to hamstring graft in terms of the cost of the ACL reconstruction. It seems reasonable then, if the clinical outcome does not differ significantly between the grafts, to considered the most inexpensive graft for ACL reconstructions due to restricted economy. Taking this into account, the femoral press-fit fixation ACL reconstruction is a very low costs consuming method comparing to other techniques, especially utilizing different fixation devices.

Similar to other techniques it has also disadvantages and limitations. Comparing for example to hamstring techniques it is not a definitely cosmetic method, what may be an issue for some patients. The poor bone quality, especially in older women might be one of the possible limitations. The two incision operation method, may be technically demanding for beginners. However in our study we did not observe any complications related neither to bone quality nor to technical site.

There is not a big number of studies presenting long-term results of ACL reconstruction [3,14,20,39]

In our study we present the 15-year results of femoral press-fit ACL reconstruction with patellar tendon autograft in a series of 52 patients. Good clinical results were documented in subjective and objective evaluation. The mean Lysholm and Tegner score were 86.3 and 6.8, respectively. These results are comparable to observations reported by others [3,4,14,35]; it is reassuring to note that these results are maintained to 15 years. Subjectively, only two patients were found to have a unstable knee and reported giving-way postoperatively.

According to the IKDC standard evaluation form, the overall improvement was documented. 66.5% of 52 patients had normal or nearly normal knee (grade A or B) at the 15-year follow-up. At the time of follow-up 42 patients (81%) had 0 or 1+ on the Lachman test. There was also a marked reduction in the degree of pivot shift after the reconstruction; 43 patients (83%) had a value of 0 or 1+. Similar results have been noted in the literature after ACL reconstruction [3,4,14,20,37]. The mean range of motion was 0° to 131°. None of our patients had a loss of extension of more than 7°. Similar results were reported by Al-Husseiny (in 3 cases loss of extension between 3° and 5°, in 2 cases loss more than 5°) [23] and Buss (in 4 cases loss of extension between 1° and 5°, in 1 case loss of 8°) [40].”

The donor site morbidity seems to be the major concern of all techniques utilizing BPTB grafts. It includes complications such as anterior knee pain, pain when kneeling, patellar fracture, patellofemoral crepitation, numbness caused by damage of the infrapatellar branch of the saphenous nerve and possible loss of quadriceps strength [14,16,36,41]. In different studies symptoms related to patellar tendon are observed in 40-60% of patients [3,4,16,20,37]; in our study it was 42%. It seems possible, like Shelbourne and Trumper [15] suggested, to decrease the incidence of anterior knee pain with modern accelerated rehabilitation programs. Fracture of the patella is a rare complication and occurs in 0.1–3% of the cases [42,43]. In this study, no patella fracture was reported. We noted two cases of arthrofibrosis defined as 10° loss of motion in the involved knee postoperatively, that needed arthroscopic arthrolysis.

The development of early osteoarthritis might be the main consequence of ACL rupture. We already know that ACL reconstruction does not prevent osteoarthritis, and the development of osteoarthritis post-ACL reconstruction has been reported for all types of reconstructions [1,3,36,44]. In our study, radiographic examination was performed on all of 52 patients at 15 years after surgery. There was a significant increase of incidence and severity of osteoarthritis between preoperative assessment and 15 years (p = 0.001). Others have reported similar incidences of degenerative changes seen on radiographs [3,4,14,20,39,44]. At the same time there was no correlation between arthritic changes and stability of the knee and subjective evaluation observed.

In conclusion, most of our patients had a satisfactory outcome after ACL reconstruction using femoral press-fit technique, and the results are comparing well to the literature. This ACL reconstruction technique, after switching to arthroscopically assisted, is still used in our centre.

We are aware of limitations associated with the present study. Limitations are related with restricted subject numbers and retrospective analysis. The small number of patients is the result of very strict criteria of inclusion. We aimed to analyze a very definite group of patients. In addition certain number of patients was lost to follow-up. Despite the limitations, in our opinion the present study allows to draw rational conclusions.

## Conclusions

Our study proved that the BPTB femoral press-fit fixation can be recommended as the fixation technique for ACL reconstruction.

The advantages of this method include unlimited bone-to-bone healing, reduction of disadvantages associated with hardware, cost effectiveness, and ease for revision surgery. In our opinion the BPTB femoral press-fit fixation technique can be safely applied in clinical practice and allows patients to return to preinjury activities also including high-risk sports.

## Competing interests

The authors declare that they have no competing interests.

## Authors’ contributions

WW – study design, data collection, manuscript preparation, data interpretation. MW – data collection, literature search. BK – data collection. SD – data interpretation. AC – statistical analysis, literature search. WT – statistical analysis, literature search. JW – data interpretation. All authors have read and approved the final manuscript

## Pre-publication history

The pre-publication history for this paper can be accessed here:

http://www.biomedcentral.com/1471-2474/13/115/prepub
